# When Guidelines Meet Reality: Frailty’s Role in Shaping Heart Failure Therapy

**DOI:** 10.1016/j.jacadv.2025.102000

**Published:** 2025-07-23

**Authors:** Muhammad Salman Sabri, Shaival Sharma, Muhammad Ahmad Sadiq, Usama Sadiq, Gregory T. Gibson, Donald Haas

**Affiliations:** a Department of Internal Medicine, Jefferson Abington Hospital, Abington, Pennsylvania, USA; b Department of Cardiology, Thomas Jefferson University Hospital, Philadelphia, Pennsylvania, USA; c Department of Cardiology, Jefferson Abington Hospital, Abington, Pennsylvania, USA; d Department of Medicine, Yale University, New Haven, Connecticut, USA

**Keywords:** angiotensin-converting enzyme, angiotensin receptor blocker, beta-blocker, cardiovascular disease, frailty, geriatric, guideline-directed medical therapy, heart failure, mineralocorticoid receptor antagonist, prevention, sodium-glucose cotransporter-2

## Frailty and its defining criteria

Frailty is characterized by heightened vulnerability to stressors and has physical, cognitive, psychosocial, and nutritional domains.^1^ According to Fried et al's widely recognized operational definition, frailty is defined as having 3 or more of the following characteristics: self-reported exhaustion, weakness (measured by decreased grip strength), slow walking speed, decreased physical activity, and unintended weight loss of more than 10 pounds within a year.^1^

## Use of guideline-directed medical therapy in the real world

The 4 main drug classes that make up guideline-directed medical therapy (GDMT) for heart failure with reduced ejection fraction (HFrEF) are beta-blockers, agents that target the renin-angiotensin-aldosterone system, such as angiotensin-converting enzyme (ACE) inhibitors, angiotensin receptor blockers (ARBs), mineralocorticoid receptor antagonists (MRAs), and sodium-glucose cotransporter-2 (SGLT2) inhibitors. In numerous large-scale randomized controlled trials (RCTs), use of these classes of medications have shown significant decreases in morbidity and mortality. However, the utilization of these medications in certain clinical settings is still quite difficult, especially when working with elderly and frail patients.

In patients with HFrEF, frailty can hinder the initiation and titration of GDMT and clinicians frequently voice concerns about tolerance, adverse effects, and challenges of polypharmacy. Thus, many patients do not receive the GDMT at doses that demonstrated benefit in clinical trials. Suboptimal use of GDMT has even been noted even in controlled clinical trial settings. For example, older patients with HFrEF were found to use GDMT less frequently in the CHAMP-HF (Change the Management of Patients with Heart Failure) and WET-HF (West Tokyo-Heart Failure) studies.^2^ Similarly, individuals aged ≥80 years had a considerably lower chance of receiving appropriate therapy compared to younger patients, according to the SwedeHF registry.^3^ According to a GUIDE-IT (The Guiding Evidence Based Therapy Using Biomarker Intensified Treatment in Heart Failure) post hoc study, patients with a significant burden of frailty had a lower chance of achieving optimal GDMT (17.7% vs 28.4%).^4^ The ESC-HF-LT (European Society of Cardiology Heart Failure Long-Term Registry) registry revealed that 34% of patients over 75 years were undertreated despite recommendations,^3^ and the FLAGSHIP (Frailty in Heart Failure: A Multicenter Prospective Cohort Study) study demonstrated that greater physical frailty independently predicted reduced GDMT use and a worse prognosis.^3^ In summary, these findings demonstrate how age and frailty have adversely impacted the implementation of GDMT in patients with HF.

## Obstacles and strategies to overcome GDMT initiation in frail patients with HF

Although frail patients with HF are known to have higher rates of adverse clinical outcomes, these may be partly driven by under prescription of GDMT, rather than frailty alone. One retrospective study^5^ using Danish registry data demonstrated that patients with moderate or severe frailty still experienced reductions in mortality and HF hospitalizations with appropriate therapy. Therefore, careful initiation and titration of GDMT, paired with close monitoring for adverse effects, should be prioritized to help reduce morbidity and hospital readmissions in this high-risk population.

Initiating and titrating GDMT in frail patients with HF is often complex and nuanced. These patients often have low normal blood pressure, and multiple comorbidities; all of which heighten their vulnerability to adverse drug effects. The burden of polypharmacy only adds to the challenge, increasing the risk for drug-drug interactions and adverse events. Additionally, clinician hesitation may be another major factor. Concerns regarding tolerability, coupled with knowledge deficit by limited exposure to recent data on GDMT in frail population leads to therapeutic inertia. Many providers also remain understandably cautious of prescribing GDMT in frail patients with HF as there is limited representation of this subgroup in landmark RCTs. On a broader level, system-related issues may also interfere with implementation. Fragmented care, poor outpatient follow-up, and limited resources in primary care settings can undermine efforts to initiate and titrate these life-saving therapies. There may be reluctance from patients to take additional medications, which may delay or limit therapy.

Despite these barriers, there are practical steps we can take. A “start low, go slow” approach remains the cornerstone. GDMT should be started at reduced doses and up titrating based on tolerance, hemodynamic stability, and stable laboratory tests. Reviewing medications to deprescribe nonessential medications is equally important. Simplifying medication regimens can open the door to further add remaining pillars of GDMT, thus providing maximum mortality and morbidity benefit while minimizing adverse effects. Fixed-dose combinations, though still an evolving option, may also help by reducing pill burden and making adherence easier. Education is key not just for patients, but also for primary care and general internal medicine providers. Sharing up-to-date evidence on the survival and symptom benefits of GDMT, along with practical tips for managing side effects, can empower more confident prescribing. It is important to provide education around manageable side effects like mild orthostatic symptoms, small increases in potassium or creatinine which are often expected and not necessarily reasons to discontinue therapy. Patients and providers should also understand that a lower blood pressure, in the absence of symptoms or signs of low cardiac output, should not automatically be a barrier to starting or continuing GDMT. Multidisciplinary care models are especially valuable here. Involving cardiologists, pharmacists, and nurse practitioners can offer the support that is required to fine-tune therapy, catch problems early, and avoid clinical inertia. There is need for GDMT RCTs inclusive of frail patients with HF using tailored protocols that test initiation of multiple agents at very low doses. Additionally, shared decision-making should be a standard part of every encounter. For many older adults, particularly those over 75 years, avoiding hospitalization and maintaining independence may take priority over extending lifespan.

## Initiation and titration of GDMT in patients with frailty

In the GUIDE-IT trial, frailty was identified not only as a barrier to the initiation of GDMT but also as an independent predictor of adverse clinical outcomes, including increased mortality and HF hospitalizations.^4^ Frail patients were less likely to be prescribed beta-blockers and ACE inhibitors or ARBs compared to nonfrail patients, although the prescription rates were similar between beta-blockers and angiotensin converting enzyme (ACEi) inhibitors/ARBs within the frail cohort. MRA were prescribed even less frequently among frail patients than beta-blockers or ACE inhibitors/ARBs. Importantly, patients who received triple therapy (beta-blocker, ACE inhibitor/ARB, and MRA) experienced fewer adverse outcomes, underscoring the importance of initiating all 4 classes of GDMT, even at low dose in frail patients, followed by gradual up titration based on tolerability and ongoing clinical and laboratory reassessment. Clinically significant adverse effects to consider during titration are postural hypotension, worsening renal function, hyperkalaemia, or symptomatic bradycardia.

**Figure 1:**
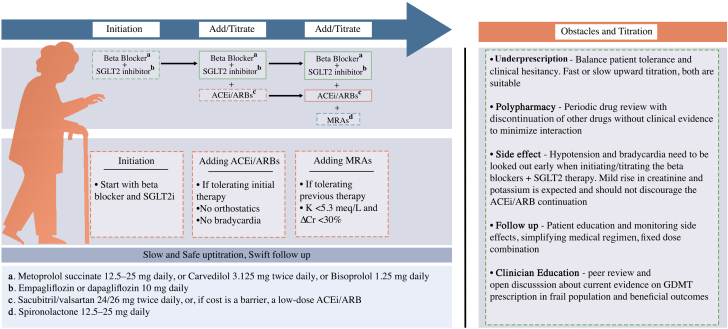
GDMT in Frail Patients: Obstacles and Strategies Provides a sequenced approach for the initiation, and titration of GDMT in frail patients with heart failure; starting with beta-blockers, and sodium-glucose cotransporter-2 inhibitors with addition of renin-angiotensin-aldosterone system antagonism inhibitors, and mineralocorticoid receptor antagonists based on tolerance and adverse effects. It provides starting doses of GDMT and escalation steps. It also highlights the barriers to initiating GDMT in frail patients with heart failure and outlines strategies to overcome them. ACEi = angiotensin-converting enzyme inhibitor; ARB = angiotensin receptor blocker; GDMT = guideline-directed medical therapy; MRA = mineralocorticoid receptor antagonist; SGLT2i = sodium-glucose cotransporter-2 inhibitor.

In landmark trial including PARADIGM-HF (Prospective comparison of ARNI with ACEI to Determine Impact on Global Mortality and morbidity in Heart Failure), the number needed to treat (NNT) in 1.5 to 2 years to prevent one death compared with placebo was 11 for angiotensin receptor–neprilysin inhibitor (ARNI), 18 for ACE inhibitors, 24 for ARBs, 8 for beta-blockers, and 15 for MRA. These findings highlight the importance of initiating beta-blocker therapy early during hospitalization, even at low doses. In cases where hypotension is a concern, a selective beta-1 blocker like metoprolol is preferred over carvedilol with combined beta-1 and alpha-1 blockade, which may have a greater impact on blood pressure. ARNI therapy has a lower NNT compared to ACE inhibitors or ARBs and should be considered in eligible patients, particularly if cost is not a barrier. However, given the lower NNT for beta-blockers compared to ARNI, beta-blockers should remain a priority in initial HF management in frail patients.^6^ In a retrospective study,^7^ patients with HF and frailty were better able to tolerate beta-blockers compared to renin-angiotensin-aldosterone system antagonists. In landmark trials, the NNT for empagliflozin and dapagliflozin was 19 over 16 months and 21 over 18 months, respectively. These NNTs are comparable to those of ACE inhibitors and ARBs, but with fewer side effects, given the minimal impact of SGLT2 inhibitors on blood pressure, renal function, and potassium levels. This favourable safety profile supports early initiation of SGLT2 inhibitors alongside beta-blockers during hospitalization, prior to introducing the remaining components of GDMT.^8^ We propose starting treatment with a low-dose beta-blocker such as metoprolol succinate 12.5 to 25 mg daily, carvedilol 3.125 mg twice daily, or bisoprolol 1.25 mg daily alongside a fixed-dose SGLT2 inhibitor (empagliflozin or dapagliflozin 10 mg daily). Patients should be followed closely to monitor for symptoms such as orthostatic hypotension or bradycardia. If these are not limiting, a low-dose renin-angiotensin system inhibitor may be added—preferably sacubitril and valsartan, 24 mg and 26 mg twice daily, or if cost is a barrier, a low-dose ACE inhibitor as an alternative (given the slightly more favourable NNT with ACEi compared to ARNI). The final agent to introduce should be a MRA, such as spironolactone 12.5 to 25 mg daily. This stepwise, low-dose approach leverages the complementary mechanisms of multiple GDMT agents while minimizing the risk of adverse effects. The key takeaways are summarized in Figure 1.

There is often clinical inertia and limited familiarity with GDMT among primary care physicians, leading to a reliance on cardiologists for initiation, particularly in frail patients However, RCTs^9^ have consistently shown that the benefits of GDMT on mortality and morbidity are preserved regardless of a patient’s frailty status. Although initiating GDMT at target doses can increase the risk of adverse events, the long-term clinical benefits remain significant. This stepwise, low-dose approach leverages the complementary mechanisms of multiple GDMT agents while minimizing the risk of adverse effects. In frail patients, initiating more classes of therapy at lower doses is likely to be safer and more effective than fewer agents at higher doses.

## Funding support and author disclosures

The authors have reported that they have no relationships relevant to the contents of this paper to disclose.
